# Pleiotropic Effects of Secretin: A Potential Drug Candidate in the Treatment of Obesity?

**DOI:** 10.3389/fendo.2021.737686

**Published:** 2021-10-04

**Authors:** Sanna Laurila, Eleni Rebelos, Miikka-Juhani Honka, Pirjo Nuutila

**Affiliations:** ^1^ Turku PET Centre, University of Turku, Turku, Finland; ^2^ Heart Center, Turku University Hospital, Turku, Finland; ^3^ Department of Cardiology, Satakunta Central Hospital, Pori, Finland; ^4^ Department of Endocrinology, Turku University Hospital, Turku, Finland

**Keywords:** secretin, obesity, gastric hormone, brown adipocyte, appetite

## Abstract

Secretin is the first hormone that has been discovered, inaugurating the era and the field of endocrinology. Despite the initial focus, the interest in its actions faded away over the decades. However, there is mounting evidence regarding the pleiotropic beneficial effects of secretin on whole-body homeostasis. In this review, we discuss the evidence from preclinical and clinical studies based on which secretin may have a role in the treatment of obesity.

## Introduction

We are currently facing a global epidemic of obesity (1). Obesity poses an additional risk for several diseases comprising cancer, neurodegeneration, cardiovascular disease (CVD), musculoskeletal disorders, and an increased vulnerability to infections (2–6). Of these CVD is the leading cause of death world-wide (7). While lifestyle modification has been shown to be only a weak arm in the battle against obesity, bariatric surgery (BS) represents today the most effective treatment to induce significant and sustained weight loss. As recently demonstrated by Yoshino et al., the beneficial metabolic effects of BS can be ascribed solely to weight loss itself, rather than to any weight-loss-independent effects (8).

It is now well-established that the beneficial effects of BS on weight loss are not only to be attributed to decreased nutrient intake, and decreased nutrient absorption, but several other mechanisms are involved, such as the marked elevation of gut-derived peptides with anorexigenic action, such as glucagon-like peptide 1 (GLP-1). GLP-1 is secreted by the L cells of the intestine in response to feeding. Apart from being an incretin hormone (thus stimulating insulin secretion after oral ingestion of nutrients), GLP-1 has important effects on regulating appetite. Semaglutide, a long-acting GLP-1 analogue, has recently shown promising results in terms of weight loss, with subjects receiving 2.4 mg of Semaglutide once weekly on top of lifestyle intervention (9).

The intestines secrete several other hormones as well. One such is secretin, which has recently gained back interest from the metabolic community. It was discovered in 1902 by Bayliss and Starling to stimulate pancreatic fluid secretion, becoming the first hormone ever discovered and inaugurating the era and the field of endocrinology. Since then it has been shown that secretin receptors are present in nearly every organ throughout the body (10). Aside from the classic exocrine effects, secretin has several interesting metabolic effects. It is a powerful lipolytic agent and its levels are increased after prolonged fasting (11). Moreover, preclinical and clinical studies have recently shown that secretin may induce satiation (12, 13). Also following bariatric surgery, secretin levels have shown to be increased (14). Taken together the gastrointestinal hormone secretin may have potential in future weight loss strategies. In this review, we describe the basic characteristics of secretin secretion and its effects on whole-body homeostasis, with special interest in its action as a satiation signal.

## Regulation of Secretin Secretion and Gastrointestinal Effects

**Figure 1:**
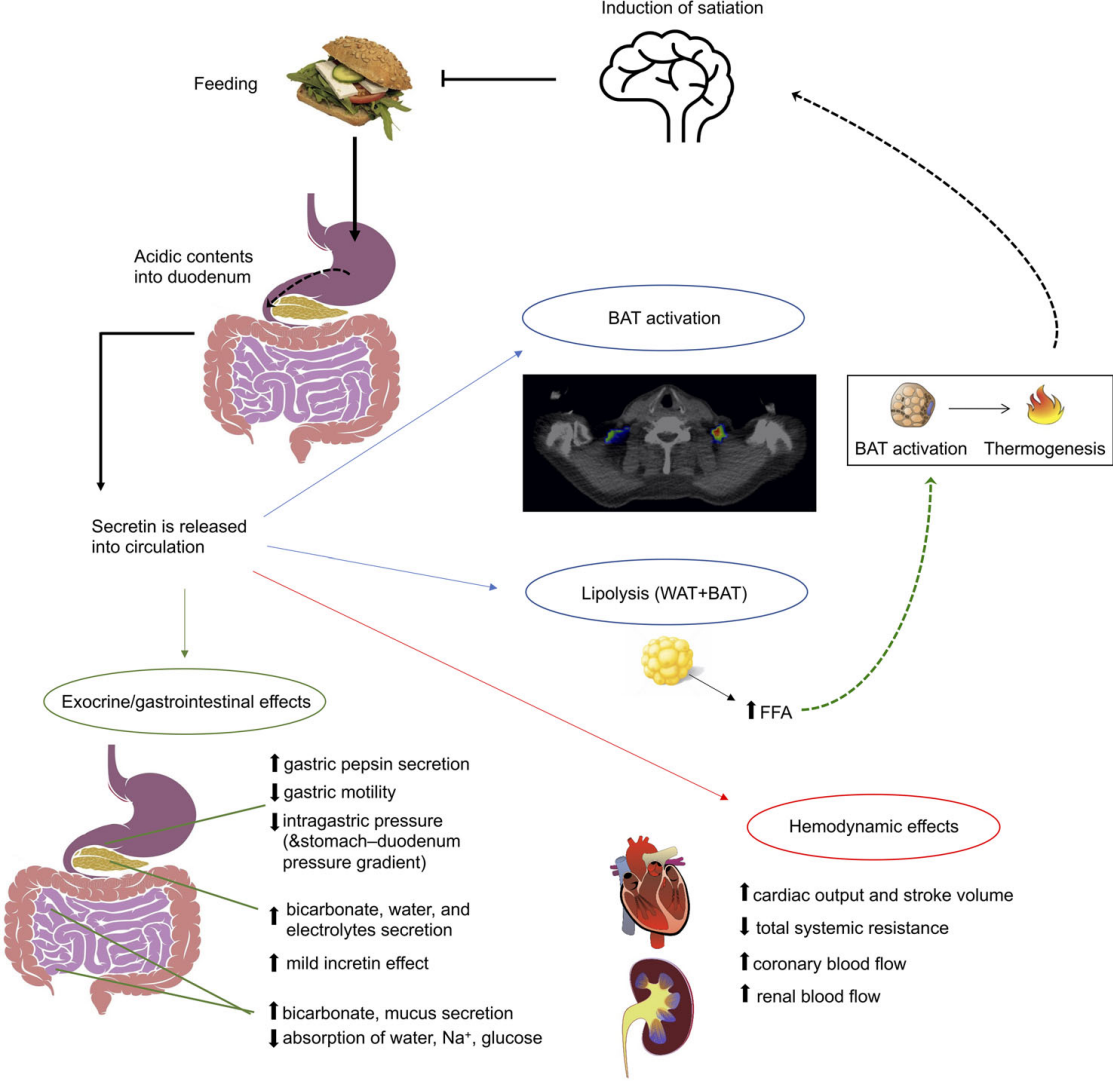
Figure summarizing the best-established effects of secretin. Apart from the well-established exocrine functions of secretin, it also has a mild incretin effect, induces appetite and brown adipose tissue (BAT) activation. Also, during prolonged fasting it enhances lipolysis. Secretin has also been shown to increase cardiac output and stroke volume.

Human secretin is synthesized as a pre-propeptide of 121 amino acid residues, containing a signal peptide (residues 1-18), propeptide (19-26), secretin (28-54), and propeptide (58-121) (15). This pre-propeptide is cleaved from both ends to achieve the active peptide of 27 amino acid residues. Secretin is predominantly synthesized by the S-cells in the crypts of Lieberkühn of the duodenal epithelium (16). Other sites with relatively high expression of secretin mRNA include intestinal enteroendocrine cells in the jejunum, ileum, colon, and rectum as well as plasmacytoid dendritic cells (17). Its release is initiated during feeding, when acidic contents of the stomach move into the duodenum (16) and duodenal pH decreases to 3 - 4.5 (18, 19) (**Figure 1**).

In rats, Green and colleagues showed that secretin secretion is larger after intraduodenal infusion of fat, compared to protein (20).Also, the fatty-acid composition of a meal induces different levels of secretin release. In a study comparing equicaloric fat emulsions, given intraduodenally in women who had undergone cholecystectomy, it was shown that whereas neutral fat did not induce any significant secretin release compared to the fasting state, medium-chain fatty acids yielded a large increase in secretin release (21). Notably, the consumption of the medium-chain fatty acid meal was followed also by a marked decrease in intraduodenal pH, whereas neutral fat modified the pH values only slightly. However, no significant differences were found in the intraduodenal pH values between the 2 groups at postprandial states. Thus, the authors proposed that even though the duodenal acidity is an important determinant of secretin release, other factors are also involved which may potentiate the secretin response, and even alter the pH threshold of secretin secretion (21). Recently, glucose was also shown to promote secretin’s release (14), even though to the best of our knowledge a direct comparison between glucose and FFA stimulation of secretin release has not been investigated. All in all, the exact mechanisms controlling secretin’s release are incompletely understood. A secretin-releasing peptide has been found to promote it, but the exact nature of this mechanism is unclear (22, 23). Pancreatic phospholipase A2 from the upper small intestine has also been found to stimulate secretin release (24, 25).

The oldest and best-known function of secretin is the stimulation of pancreatic exocrine secretion (10). This is also initiated by vasoactive intestinal peptide (VIP), cholecystokinin (CCK) and vagal stimulation (10). Secretin also neutralizes the acidic contents of the duodenal lumen by stimulating pancreatic acinar cells and duodenal Brunner’s glands to produce bicarbonate and water (26, 27), and by inhibiting gastric acid secretion and gastric motility (10). The intestinal lining is protected by these effects, while digestive enzymes start to break down nutrients (10). In addition to ingested nutrients, pancreatic proteases also break the secretin releasing peptide, creating a negative feedback loop for secretin release (23). Secretin also induces biliary secretion of water, bicarbonate and chloride (28), but not bile acids (29), while inhibiting the absorption of water, sodium, and glucose in the jejunum and ileum (30–33).

## Pharmacokinetics

Secretin has a relatively short half-life in plasma in humans (2.5-4.0 min) (34–37). Animal studies have revealed that secretin removal from the circulation occurs mainly through the kidneys (38) but also the capillary beds of various other tissues (39, 40). Despite the kidneys being an important organ for secretin removal, only small amounts of secretin appear in the urine (41) because, after glomerular filtration, secretin is reabsorbed in the distal part of the nephron (42, 43). Further research is required to understand the pharmacokinetics of secretin in humans and its molecular mechanisms.

## Current Clinical Uses

Currently, synthetic secretin is in clinical use solely for rare and specific diagnostic purposes. In Zollinger-Ellison syndrome, a rare neuroendocrine tumour (gastrinoma) produces high levels of gastrin (44), leading to abnormally increased gastric acid production. A secretin stimulation test can be performed, if a gastrinoma is suspected but gastric pH and serum gastrin levels are not diagnostic. After an overnight fast, a bolus of secretin is given (2 IU/kg) intravenously, and serum gastrin levels are measured at 0, 2, 5, 10 and 15 minutes. Even though in normal subjects, secretin inhibits gastrin release (45), it stimulates the gastrinoma cells to release gastrin, which leads to a significant increase in serum gastrin levels. Serum gastrin levels greater than 200 pg/ml are diagnostic. Secretin is sometimes also used to investigate exocrine pancreatic insufficiency (46, 47). It can be given during magnetic resonance cholangiopancreatography, in order to study pancreatic exocrine function, or to evaluate the anatomy of the pancreatic duct (48).

## Hemodynamic Effects of Secretin

Early studies have shown that pharmacological doses of secretin increase renal blood flow in healthy humans by 58% (49), and subsequently in patients with angina and heart failure (NYHA class III-IV) it was shown that a secretin infusion significantly increases cardiac output (~20%) and stroke volume (50, 51). Systemic resistance was decreased, while heart rate was not affected. These effects are indicative of a vasodilator effect of secretin, whereas an inotropic effect of secretin is also likely (50). Our group is currently investigating whether secretin has effects on myocardial metabolism and renal function on healthy humans, assessed with [^18^F]-FDG-PET (NCT03290846).

## Effects of Secretin in the Lungs

In addition to the gastrointestinal tract, the secretin receptor is abundantly expressed in the distal regions of the lungs (52), specifically in type 2 alveolar cells (17) that are responsible for surfactant secretion (53). In addition, some secretin receptor expression is present in the club cells of the bronchiolar epithelium (17), and tertiary bronchial smooth muscle (52). It is likely that secretin participates in maintaining the airway surface liquid and mucociliary clearance, and bronchial smooth muscle relaxation (52).

## Secretin as a Neuropeptide

The potential central effects of secretin were first recognized when secretin-like bioactivity was found in porcine brain extracts (54). A study on human brains found secretin immunoreactivity in the pyramidal neurons of the motor cortex, deep cerebellar nuclei, cerebellar Purkinje cells and the hippocampal and amygdala nuclei (55). Spatially, the secretin receptor is even more widely distributed than its ligand, which may indicate that several different neuronal functions could be modulated by secretin (56). Secretin may even be important in early postnatal neurological development. In secretin deficient mice, hippocampal neurogenesis was disturbed, which lead to impaired neurobehavioral development (57). Secretin deficiency also led to impaired synaptic plasticity in the hippocampus (58).

Secretin could also have wide effects on the autonomic nervous system, since it has been shown to have regulatory effects on catecholamine metabolism in the axon terminals of sympathetic nerves (59). It also has a stimulatory effect on cyclic adenosine monophosphate (cAMP) production (60). cAMP regulates the enzyme tyrosine hydroxylase (61), which catalyses the rate limiting step of catecholamine biosynthesis. Secretin has been shown to increase tyrosine hydroxylase activity in the sympathetic ganglia and several autonomic end organs (62). When rats were given an interventricular infusion of secretin, there was an increase in tyrosine hydroxylase activity in the hypothalamus (63).

Rodent studies indicate that secretin is also involved in the regulation of dihydroxyphenylalanine (DOPA) synthesis and turnover (64). Secretin also facilitates gamma-aminobutyric acid, or GABAergic input of Purkinje cells in the cerebellum (65, 66) and vasopressin expression and release in the hypothalamus (64). However, this effect on both vasopressin and oxytocin release may also be through a noradrenergic pathway, as shown in a rat model by Velmurugan et al. (67). All in all, it has been proposed, that the central actions of secretin may be related to fluid homeostasis (68, 69), food intake (70) and control of social behaviour (71, 72). These effects by peripherally secreted secretin would be mediated through the autonomic nervous system (ANS) (70, 73), or directly after transmembrane diffusion of the hormone (74).

## Fluid Homeostasis

Initially it seemed that secretin had a diuretic effect on dogs and humans (75), but there were opposite findings in rats (76). Secretin increases renal blood flow (77), and glomerular filtration rate and glomerular plasma flow are also increased in dogs (78). More recent studies in mice showed that secretin stimulates vasopressin expression and release in the hypothalamus (68), and also increases renal water absorption through a vasopressin-independent mechanism on aquaporin 2 channels in the collecting tubules in hyperosmolar conditions (79). Centrally injected secretin induced water drinking behaviour in rats, which increased diuresis, while peripherally injected secretin did not have this effect (69). Thus, it is likely that secretin’s effect on fluid homeostasis varies depending on whether its effect is peripheral or central, or depending on conditions such as osmolarity or serum secretin concentration.

## Appetite Control

Another suggested central effect of secretin is appetite control (73). In an elegant study, Yang and colleagues demonstrated that an intraperitoneal injection of secretin induces a dose-dependent increase in the number of Fos-positive neurons in the arcuate nucleus, the hypothalamic nucleus that suppresses appetite (73). Subsequently it was shown that both peripheral and central administration of secretin suppresses appetite in mice (80), and that following either vagotomy, or administration of capsaicin, an afferent neurotoxic agent, the anorexigenic effects of secretin were attenuated (70).

## Insulin Secretion and Glucose Homeostasis

Secretin has a mild incretin effect, but this effect is much smaller than that of GLP-1 and GIP. Early studies showed that insulin secretion was increased by secretin during a glucose infusion and pre-treating patients with secretin also potentiated glucose-stimulated insulin release (81). The increase in insulin levels was small and only lasted a few minutes, due to which the authors suggested that secretin only stimulates the first phase of insulin release and not production (82).

In another study on healthy subjects, a physiological dose of intravenous secretin (0.5 pmol/kg) did not induce an increase in insulin secretion, whereas pharmacologic doses of secretin (16 pmol/kg) induced a significant increase in plasma insulin concentrations, which returned to pre-stimulus values after 20 minutes (83). Isoprotenerol and secretin-induced insulin release was blunted in the same effect in adult-onset diabetics, compared to healthy controls (84).

## Secretin and Lipolysis

Secretin receptors induce lipolysis in white adipose tissue, initiated by its ligand (85, 86). This happens through Gs-coupled cAMP - protein kinase A (PKA) signalling, independently of sympathetic activation (87). During prolonged fasting, plasma secretin levels are increased almost 8-fold from day 1 to 3 (11, 88–90). These levels are much higher than the levels achieved through feeding and supports secretin’s role as a potent lipolytic agent (85, 86). The mechanism by which the increased secretin levels are achieved is not known but it is independent of hydrochloric acid concentration (88, 91). Secretin levels have also been studied during exercise when lipolysis is also increased. A 3 hour bicycle exercise intervention markedly increased serum secretin levels, both during exercise and after 3 hours of rest (92). O’Connor et al. investigated marathon runners and found that secretin levels were increased along with all other examined gastrointestinal peptides, except for insulin both immediately and 30 minutes after finishing the race (93). At the time, no direct speculation was made on the mechanism or purpose. It was previously unknown what effect secretin has on brown adipose tissue (BAT). Since lipolysis is important not only in fuelling, but also initiating uncoupling protein 1 (UCP1) thermogenesis in BAT, studies on secretin as a BAT activator seemed warranted.

## Secretin and Obesity

There is evidence that the increase of serum secretin in prolonged fasting is blunted in obesity. In a study conducted by Andrews et al., gastric hormone levels were measured after 12 and 36 hours of fasting and after an oral glucose tolerance test (OGTT) (94). Secretin, glucagon, and vasoactive intestinal polypeptide (VIP) increased in lean but not obese after 36 h of fasting (94). Further, obese subjects had an insulin secretion response to a smaller dose of secretin than lean ones, even if the response to a higher dose was similar in groups (94). Potential differences in fasting and postprandial secretin levels in lean and obese subjects have not been thoroughly investigated, but a small study by Vezina et al. reported no difference in the fasting and postprandial secretin levels after ingestion of a small volume liquid fatty meal to promote gallbladder emptying, between lean and obese subjects (95).

Expression of the secretin receptor may also be affected by obesity, as a positive correlation between BMI and ApoB levels with the SCT receptor expression in omental fat in humans has been described (96).

## Secretin and Bariatric Surgery

In a study by Miskowiak et al. in 1984, 11 morbidly obese patients underwent gastroplasty and plasma secretin levels were measured before and 3 months after (97). Postprandial secretin levels were higher after gastroplasty compared to before the operation, but the difference was not statistically significant, which could be either due to the small sample size (n=11) or the operation technique (97). Interestingly, a recent study by Modvig et al. noted a two- to threefold increase in postprandial secretin three months after RYGB (14). Nergård et al. also noted a two- to threefold increase in postprandial secretin three months after RYGB (98).

The increase in postprandial secretin levels is in line with a finding in rats that underwent RYGB, where secretin was found to be upregulated after the operation in the alimentary limb and proximal common channel (99). Modvig et al. showed with a rat model, that there are glucose sensitive S-cells in the distal part of the small intestine, which could explain the increase in postprandial secretin after RYGB (14). In humans, the results are somewhat conflicting. Nergård et al. found no increase of secretin secreting cells in the perianastomotic jejunum in 18 patients 12 months after RYGB (98). In a study by Rhee et al, mucosal biopsies were collected from the small intestine during surgery and 10 months after RYGB (100). Immunohistochemistry and RNA sequencing results from different biopsy sites were compared in 12 patients with T2D and 11 healthy subjects. Secretin encoding SCT was reduced significantly in all biopsy sites (alimentary limb, secretory limb and common limb), except the alimentary limb of the non-diabetic group (100). Taken together, even though most clinical studies suggest an increase in circulating secretin levels following bariatric surgery, the results regarding the expression of the secretin receptor are conflicting. Therefore, more studies are needed in order to clarify this and also to address whether some of the beneficial effects following bariatric surgery may be attributed to changes in secretin levels.

## Secretin: A Novel Mediator of an Appetite Controlling Gut-BAT-Brain Axis

**Figure 2:**
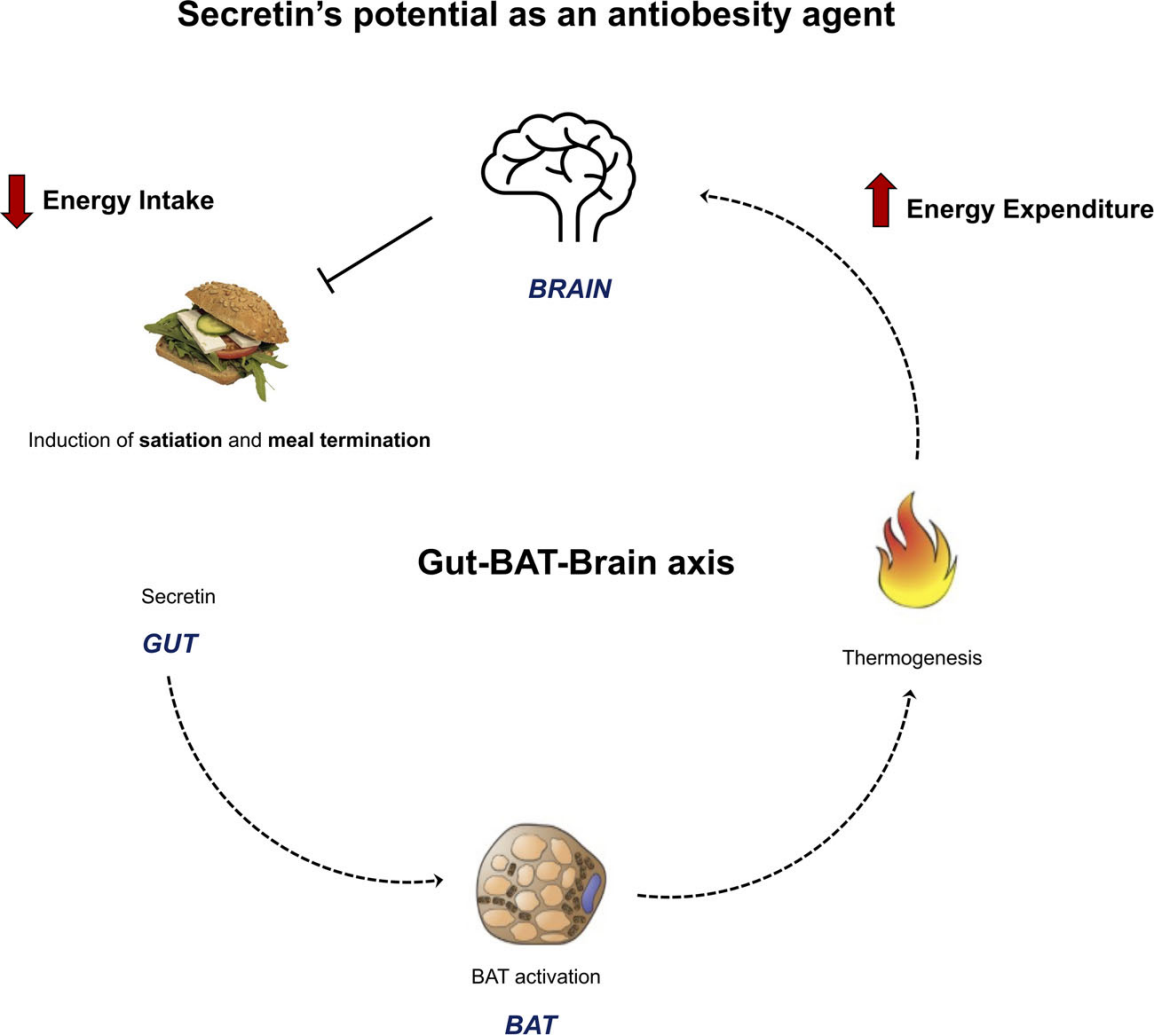
Figure summarizing the novel anti-obesity effects of secretin. Li et al., 2018 showed the presence of a gut – BAT – brain -axis with rodent models. Secretin, secreted by the gut during feeding, binds to secretin receptors in brown adipose cells. This induces thermogenesis, which functions as a satiation signal for the brain and terminates feeding. Secretin’s BAT activation and satiation effects were shown to translate to humans by Laurila et al., 2021. These results highlight that secretin has a rare dual role on energy homeostasis, potentially both increasing energy expenditure and decreasing energy intake. This makes it an attractive anti-obesity agent for further studies.

It was recently shown with *in vitro* and *in vivo* experiments, that secretin has a thermogenic effect on BAT (12) (**Figure 2**). Secretin activated thermogenesis in a culture of adherent primary brown adipocytes, an effect which was much stronger (~50-fold) than that of isoproterenol, a β-adrenergic receptor agonist (12). The thermogenic effect of secretin was independent of activation of the adrenergic receptors, since pre-treatment of brown adipocytes with propranolol did not affect secretin-stimulated respiration, while blocking isoproterenol-stimulated respiration (12). Secretin stimulation resulted in a dose-dependent increase of cytosolic cAMP (12). Next, the thermogenic effect of secretin was also confirmed *in vivo* in mice with the utilization of indirect calorimetry and multispectral optoacoustic tomography. The former measures gas exchanges, whereas the latter detects the spectra of oxygenated and deoxygenated haemoglobin. It was shown that relative oxygen saturation was markedly decreased following secretin administration (12).

While it had already been shown that peripheral and central administration of secretin reduces food intake in fasted mice (80), the exact mechanism for this effect was not elucidated. To explore whether the satiety-inducing effect of secretin was through BAT activation, UCP1 wild type and knockout (KO) mice were studied. UCP1 KO mice did not reduce their food intake following secretin administration, confirming that this effect of secretin is mediated through BAT activation (12). However, this effect is limited to only an initial phase of feeding, possibly because of the short half-life of the peptide. Furthermore, when endogenous secretin was neutralized by an antibody, meal size and duration was significantly increased compared to controls (12). These results confirmed that the satiation effect is induced by secretin and BAT thermogenesis (**Figure 2**). The neurobiological basis of the effect was then studied with hypothalamic samples, collected from fasted WT and UCP1 KO mice after intraperitoneal injections of secretin. Secretin increased proopiomelanocortin (POMC) and decreased agouti-related protein (AgRP) mRNA levels in WT mice (12). POMC neurons have anorexigenic effects, while AgRP have orexigenic effects. Furthermore, temperature sensitive ion channels; transient receptor potential vallinoid 1 (TRPV1) were upregulated in the POMC neurons of WT mice, supporting the hypothesis of heat, generated by BAT, functioning as an appetite reducing message to the central nervous system (12).

## Recent PET and fMRI Data

We have recently conducted an imaging study that further highlights the potential of secretin as an anti-obesity agent in humans (**Figure 2**). Positron emission tomography (PET) represents the state-of-the art method for quantifying metabolic rates *in vivo* in humans. Based on the tracer used, different metabolic aspects can be evaluated (101–104). [^18^F]-FDG-PET is considered the gold standard method for studying BAT activation in humans (105), while perfusion by [^15^O]-H_2_O-PET is considered an indirect measure of BAT thermogenesis, because it has been shown to associate with BAT oxygen consumption (106).

We have recently conducted a study where secretin’s effects were investigated with whole body [^18^F]-FDG-PET, to measure glucose uptake rates, and [^15^O]-H_2_O-PET, to measure BAT perfusion. In accordance with our previous findings in mice, we showed that secretin activates BAT in healthy lean men (13). More specifically, secretin induced an increase in [^18^F]-FDG uptake in BAT, suggestive of increased metabolic activity, while perfusion was not changed in an acute setting (13). In mice, maximal thermogenesis was achieved 20 minutes after secretin administration (12). The [^18^F]-FDG scan was initiated 20 minutes after the first 1 IU/kg injection of secretin, simultaneously with another 1 IU/kg 2-minute infusion of secretin. In contrast, perfusion was measured only two minutes after the first secretin infusion (13). Since secretin stimulates BAT endogenously instead of through a faster neuronally mediated effect, the perfusion scan was likely conducted too early to measure secretin’s effect on BAT perfusion.

Evidence of a catabolic effect was found with indirect calorimetry: whole body energy expenditure increased by secretin compared to placebo (13). BAT computer tomography radiodensity was increased at the end of the scan, compared to the start, and this increase was associated with whole body fat oxidation after secretin infusion (13). Higher adipose tissue radiodensity indicates reduced intracellular triglycerides or increased perfusion (106) and as such, the results could indicate that secretin induces BAT fatty acid oxidation.

In a subsequent study, we showed that secretin administration changed the brain’s responses (as measured by the blood-oxygen-level-dependent signal by functional magnetic resonance imaging) to palatable *vs* non-palatable food cues (13). In the placebo condition, visual exposure to palatable versus non-palatable foods increased hemodynamic brain activity in the medial frontal cortex, cingulate cortex, caudate and middle and posterior insula, while this anticipatory reward-sensitive coding of the food images was abolished after secretin infusion (13). Satiety was assessed with the composite satiety score using visual analogue scale questions. We found that secretin increased the subjective satiety compared to placebo in fasting conditions and during early feeding, but this effect was no longer significant in the postprandial evaluation (13). Since subjects were instructed to feed until satiated, postprandial differences in satiety were not expected. Meal consumption following secretin was not statistically significantly decreased compared to placebo, but our study was underpowered to explore this endpoint. However, secretin delayed resumption to eat after the test meal, with a mean delay of 39 minutes, as compared to placebo (13).

All in all, our results indicate that the gut – BAT – brain axis previously shown in mice, translates to healthy, normal weight humans (**Figure 2**). Secretin activates BAT and increases whole body energy expenditure in humans, making it a catabolic agent. It also has an attenuating effect on anticipatory reward responses to appetizing food, increases satiation pre-prandially and in early feeding and delays resumption to eat. These results highlight that secretin has a rare dual role on energy homeostasis in humans, potentially both increasing energy expenditure and decreasing energy intake.

## Conclusions and Future Directions

Despite enormous efforts from several metabolic units around the globe, a complete and long-lasting resolution of obesity relies predominantly on bariatric surgery. However, BS is an invasive procedure, which is not widely and equally available around the globe. Many patients do not represent good candidates for undergoing BS and some do not wish to undergo BS. Also, even though the safety of BS has been proven, some “bariatric” patients may suffer from post-prandial hypoglycaemia (mild, moderate, or severe) (107), nutritional deficits, or gastrointestinal occlusions that need emergency treatment. Thus, identifying pathways that lead to obesity (appetite dysregulation, or decreased thermogenesis) for medical treatments of obesity is taking on a new urgency.

In this review we have highlighted secretin’s pleiotropic and somewhat forgotten metabolic roles. Most importantly, our recent findings show that secretin both increases energy expenditure and reduces appetite, making it a potential anti-obesity agent. The problem with only aiming to increase energy expenditure as a weight loss treatment is that increased energy expenditure leads to increased energy intake (108). Secretin’s dual effect on energy homeostasis makes it a very attractive candidate for future studies. Still, much work is warranted to investigate the potential of secretin as a weight-reducing agent. Our volunteers were all healthy, normal weight and male. Whether this gut-BAT-brain axis is preserved in overweight and obese individuals needs to be demonstrated. In our study, we infused intravenously a synthetic human secretin with a rapid half-life. Currently, there is no long-acting secretin analogue for human use, but it would be warranted to investigate whether a longer acting secretin analogue could provide similar or even more pronounced effects. The serum secretin levels measured in our study after intravenous infusions were similar to postprandial secretin levels. Supra-physiological levels of secretin could have a more pronounced effect on appetite and energy expenditure. Furthermore, larger clinical trials are needed in order to confirm pre-prandially administered secretin’s potential in reducing energy intake in humans. All in all, further clinical trials on secretin are warranted, as it seems to have a dual effect on energy homeostasis and could have potential in weight control.

## Author Contributions

SL, ER, and M-JH drafted the manuscript. PN critically revised the text. All authors contributed to the article and approved the submitted version.

## Funding

The study was conducted within the Centre of Excellence into Cardiovascular and Metabolic Diseases supported by the Academy of Finland (grant no. 307402), University of Turku, Åbo Akademi University; and funded by the Instrumentarium Science Foundation (grant no. 190014) (SL), The Paulo Foundation (SL), Turku University Hospital Foundation (SL) and The Finnish Medical Foundation (grant no. 2985) (SL).

## Conflict of Interest

The authors declare that the research was conducted in the absence of any commercial or financial relationships that could be construed as a potential conflict of interest.

## Publisher’s Note

All claims expressed in this article are solely those of the authors and do not necessarily represent those of their affiliated organizations, or those of the publisher, the editors and the reviewers. Any product that may be evaluated in this article, or claim that may be made by its manufacturer, is not guaranteed or endorsed by the publisher.

## Abbreviations

BAT, brown adipose tissue; BS, bariatric surgery; CNS, central nervous system; CSS, composite satiety score; CVD, cardiovascular disease; FFA, free fatty acids; fMRI, functional magnetic resonance imaging; GI, gastrointestinal; GLP-1, glucagon-like peptide 1; PET, positron emission tomography; TRPV1, Transient receptor potential vanilloid; UCP1, uncoupling protein 1; VAS, visual analogue scale.
